# Supplementary material for: Effect of 10-Day Treatment with 50 mg Prednisolone Once-Daily on Haemostasis in Healthy Men—A Randomised Placebo-Controlled Trial
Source: Biomedicines. 2023 Jul 21;11(7):2052. doi: 10.3390/biomedicines11072052 (PMC10377059; doi:10.3390/biomedicines11072052)
Supplement: Supplementary file 1 [file biomedicines-11-02052-s001.zip › biomedicines-2493846-supplementary.pdf]

# SUPPLEMENTAL FILE

## Effect of 10-Day Treatment with 50 mg Prednisolone Once-Daily on Haemostasis in Healthy Men—A Randomised Placebo-Controlled Trial

**Peter Kamstrup [1], Ema Rastoder [1], Pernille Høgh Hellmann [2], Pradeesh Sivapalan [1], Emil List Larsen [3], Jørgen Vestbo [4,5], Charlotte Suppli Ulrik [6,7], Jens P. Goetze [3,8], Filip Krag Knop [2,7,9,*] and Jens Ulrik Stæhr Jensen [1,7,*]**

[1] Section of Respiratory Medicine, Department of Medicine, Copenhagen University Hospital—Herlev and Gentofte, 2900 Hellerup, Denmark; peter.kamstrup@regionh.dk (P.K.)
[2] Center for Clinical Metabolic Research, Department of Medicine, Gentofte Hospital, University of Copenhagen, 2900 Hellerup, Denmark
[3] Department of Clinical Biochemistry, Copenhagen University Hospital—Rigshospitalet, 2100 Copenhagen, Denmark
[4] Allergi og Lungeklinikken Vanløse, 2720 Vanløse, Denmark
[5] Division of Infection, Immunity and Respiratory Medicine, School of Biological Sciences, The University of Manchester, Manchester Academic Health Science Centre, Manchester M13 9PL, UK
[6] Department of Respiratory Medicine, Copenhagen University Hospital—Hvidovre, 2650 Hvidovre, Denmark
[7] Department of Clinical Medicine, Faculty of Health and Medical Sciences, University of Copenhagen, 2200 Copenhagen, Denmark
[8] Department of Biomedical Sciences, Faculty of Health and Medical Sciences, University of Copenhagen, 2200 Copenhagen, Denmark
[9] Steno Diabetes Center Copenhagen, 2730 Herlev, Denmark
* Correspondence: filip.krag.knop.01@regionh.dk (F.K.K.); jens.ulrik.jensen@regionh.dk (J.U.S.J.)

**Table S1: Results from analyses done for curcumin versus curcumin-placebo in the prednisolone-treated group.**

| Primary outcome | Baseline (95% CI) | ΔPrednisolone+Placebo (95% CI) | ΔPrednisolone+Curcumin (95% CI) | Placebo / curcumin, n |
|---|---|---|---|---|
| TEG:MA (mm) | 61.3 (59.43, 63.17, p<.0001) | 0.61 (-0.83, 2.06, p=0.38) | -2.00 (-4.91, 0.92, p=0.16) | 11 / 4 |
| **Secondary outcomes** | | | | |
| TEG:R (min) | 5.19 (4.19, 6.18, p<.0001) | 0.13 (-0.96, 1.22, p=0.80) | -1.06 (-2.52, 0.40, p=0.14) | 11 / 4 |
| TEG:Angle (Degrees) | 63.94 (60.49, 67.39, p<.0001) | 1.26 (-1.61, 4.12, p=0.36) | -0.31 (-5.42, 4.80, p=0.90) | 11 / 4 |
| TEG:K (min) | 1.88 (1.55, 2.21, p<.0001) | -0.11 (-0.35, 0.14, p=0.37) | -0.0047 (-0.42, 0.41, p=0.98) | 11 / 4 |
| TEG:LY30 (%) | 0.39 (0.15, 0.64, p=0.0041) | 0.11 (-0.86, 1.07, p=0.81) | 1.17 (-0.74, 3.09, p=0.21) | 11 / 4 |
| Platelet count (x$10^9$/L) | 206.75 (185.29, 228.21, p<.0001) | 24.92 (9.97, 39.86, p=0.002) | -13.73 (-38.92, 11.46, p=0.26) | 11 / 5 |
| VWF:Ag (kIU/L) | 1.07 (0.85, 1.30, p<.0001) | 0.28, (0.12, 0.45, p=0.0019) | -0.075 (-0.38, 0.23, p=0.61) | 12 / 5 |
| VWF:RCo (kIU/L) | 0.78 (0.63, 0.93, p<.0001) | 0.13 (0.06, 0.20, p=0.0015) | -0.02 (-0.13, 0.10, p=0.75) | 12 / 5 |
| INR | 1.08 (1.014, 1.15, p<.0001) | 0.02 (-0.022, 0.062, p=0.32) | -0.04 (-0.12, 0.037, p=0.26) | 10 / 4 |
| APTT (Seconds) | 27.06 (25.12, 29.00, p<.0001) | -1.29 (-5.73, 3.15, p=0.54) | 3.91 (-5.07, 12.90, p=0.36) | 10 / 3 |
| Prothrombin (kIU/L) | 0.98 (0.93, 1.03, p<.0001) | 0.059 (0.0065, 0.11, p=0.029) | 0.0011 (-0.090, 0.093, p=0.98) | 11 / 5 |
| Fibrinogen (µmol/L) | 6.93 (5.12, 7.74, p<.0001) | -0.85 (-1.45, -0.25, p=0.0082) | -0.14 (-1.05, 0.78, p=0.75) | 12 / 5 |
| D-dimer (mg FEU/L) | 0.24 (0.17, 0.31, p<.0001) | -0.031 (-0.11, 0.048, p= 0.38) | -0.0054 (-0.062, 0.051, p=0.84) | 11 / 5 |
| Antithrombin (kIU/L) | 0.95 (0.90, 1.00, p<.0001) | 0.074 (0.03, 0.12, p=0.0027) | -0.035 (-0.11, 0.042, p=0.036) | 11 / 4 |
| Protein C (kIU/L) | 0.98 (0.85, 1.11, p<.0001) | 0.23 (0.10, 0.35, p=0.0013) | 0.19 (-0.0093, 0.40, p=0.060) | 11 / 4 |

Table 2. Results from the linear mixed models in the prednisolone group, investigating effect of curcumin. CI, confidence interval; TEG, thromboelastography; MA, maximal amplitude of clot; VWF:Ag, von Willebrand factor antigen; VWF:RCo, von Willebrand factor-ristocetin cofactor activatity; kIU/L, kilo international units per litre; R, reaction time; LY30,:lysis in 30 minutes; K, clotting time; FEU, fibrin equivalent units.
